# Return-to-work, disabilities and occupational health in the age of COVID-19

**DOI:** 10.5271/sjweh.3960

**Published:** 2021-06-29

**Authors:** Diane Godeau, Audrey Petit, Isabelle Richard, Yves Roquelaure, Alexis Descatha

**Affiliations:** Université Paris-Saclay, UVSQ, Univ. Paris-Sud, Inserm, Équipe d’Épidémiologie Respiratoire Intégrative, CESP, 94807, Villejuif, France; AP-HP (“Assistance Publique-Hôpitaux de Paris”), Hôpitaux universitaires Paris Seine-Saint-Denis, Hôpital Avicenne, Unité des pathologies professionnelles et environnementales, F-93009 Bobigny cedex, France; Université Sorbonne Paris Nord, F-93206 Saint-Denis, France; Univ Angers, CHU Angers, Univ Rennes, Inserm, EHESP, Irset (Institut de recherche en santé, environnement et travail) - UMR_S 1085, F-49000 Angers, France; CHU Angers, Poisoning Control Center- Clinical Data Center, F-49000 Angers, France

We have read with great interest the two editorials by Burdorf et al: “The COVID-19 pandemic: one year later – an occupational perspective” (1) and “The COVID-19 (Coronavirus) pandemic: consequences for occupational health” (2). The authors highlight the importance of the societal consequences of the outbreak and changes in the world of work to manage occupational health. The key points identified – such as individual socio­economic factors, psychological effects and occupations with highest risk of contamination – modify return-to-work approaches.

It is estimated that around 800 million people of working age worldwide were living with disabilities before the SARS-CoV-2 pandemic. In early January 2021, the cumulative COVID-19 hospitalisation rate reached 207.4/100 000 (18–49-year-olds) and 505.7/100 000 (50–64-year-olds), respectively, in the United States (3). In France, the hospitalisation rate was 411.5/100 000 across all ages (4). A recent cohort study of working-age men who were hospitalised for COVID-19 highlighted the long-term health consequences of such a disease (5).

The SARS-CoV-2 pandemic creates new challenges for occupational health, shifting attention away from return-to-work after health problems to resuming work during an outbreak, dealing with lockdown, and taking special account of workers with vulnerabilities (6, 7).

We recommend considering three different aspects of occupational medicine during a pandemic. Firstly, for most workers at high-risk of severe COVID-19, the issues of work disability and resuming work had never occurred before the epidemic. Recommendations such as physical and social distancing and wearing a facemask are highly advisable to protect against infection but may not be enough to enable some individuals to resume work. Therefore, decision-making requires individual comprehensive assessments of the underlying medical condition, the SARS-CoV-2 contamination risk associated with either regular work or teleworking, and vaccination opportunities.

The second situation concerns workers who have suffered from COVID-19. Preliminary studies suggest that long recovery duration is related to high severity (7), but this is still a matter of debate for patients suffering from “long COVID-19” (5, 8, 9), a condition for which the long-term effects remain unknown. Any long-running recovery must be considered to be a potential sign of long COVID-19. These long-lasting syndromes occur among patients with severe symptoms but have also been reported independently of acute phase severity, hospitalisation and receiving medical oxygen (8, 9). Researchers worldwide are currently investigating such syndromes. Strategies promoting return to work for these workers will need to be implemented and could be similar to programmes developed for other chronic conditions. Moreover, numerous more serious sequelae following critical illness suggest the need for enhanced support by rehabilitation and occupational health specialists.

Finally, the consequences of the epidemic must be evaluated over time for people who suffered from functional limitations before COVID-19 as their physical and mental condition may be modified by the epidemic and, specifically, the consequences of lockdown (10).

In all of these situations, medical, social, financial and working contexts are key elements. In addition to a medical assessment, the use of scales such as the Work Ability Index (WAI) (11) or the Work Productivity and Activity Impairment (WPAI) (12) can help perform long-term follow-up and provide information about work capacity and workload. It also gives a “back to basics” perspective, urging politicians to move towards a ‘decent-work-for-all’ policy, as advocated by the United Nation’s Sustainable Development Goal (SDG) 8, which the WHO has endorsed (13).
